# Referred Pain Patterns Provoked on Intra-Pelvic Structures among Women with and without Chronic Pelvic Pain: A Descriptive Study

**DOI:** 10.1371/journal.pone.0119542

**Published:** 2015-03-20

**Authors:** Thomas Torstensson, Stephen Butler, Anne Lindgren, Magnus Peterson, Margaretha Eriksson, Per Kristiansson

**Affiliations:** 1 Department of Public Health and Caring Sciences, Uppsala University, Uppsala, Sweden; 2 Department of Physiotherapy, Sundsvall Hospital, Sundsvall, Sweden; 3 Pain Center, Uppsala University Hospital, Uppsala, Sweden; University of Texas at Dallas, UNITED STATES

## Abstract

**Objectives:**

To describe referred pain patterns provoked from intra-pelvic structures in women with chronic pelvic pain (CPP) persisting after childbirth with the purpose to improve diagnostics and give implications for treatment.

**Materials and Methods:**

In this descriptive and comparative study 36 parous women with CPP were recruited from a physiotherapy department waiting list and by advertisements in newspapers. A control group of 29 parous women without CPP was consecutively assessed for eligibility from a midwifery surgery. Inclusion criterion for CPP was: moderate pain in the sacral region persisting at least six months after childbirth confirmed by pelvic pain provocation tests. Exclusion criteria in groups with and without CPP were: persistent back or pelvic pain with onset prior to pregnancy, previous back surgery and positive neurological signs. Pain was provoked by palpation of 13 predetermined intra-pelvic anatomical landmarks. The referred pain distribution was expressed in pain drawings and described in pain maps and calculated referred pain areas.

**Results:**

Pain provoked by palpation of the posterior intra-pelvic landmarks was mostly referred to the sacral region and pain provoked by palpation of the ischial and pubic bones was mostly referred to the groin and pubic regions, with or without pain referred down the ipsilateral leg. The average pain distribution area provoked by palpation of all 13 anatomical landmarks was 30.3 mm^²^ (19.2 to 53.7) in women with CPP as compared to 3.2 mm^²^ (1.0 to 5.1) in women without CPP, p< 0.0001.

**Conclusions:**

Referred pain patterns provoked from intra-pelvic landmarks in women with CPP are consistent with sclerotomal sensory innervation. Magnification of referred pain patterns indicates allodynia and central sensitization. The results suggest that pain mapping can be used to evaluate and confirm the pain experience among women with CPP and contribute to diagnosis.

## Introduction

Chronic pelvic pain (CPP) is a major therapeutic challenge for healthcare providers such as general practitioners, physiotherapists and specialized physicians and it is often difficult to reach a definitive diagnosis [1,2]. Symptoms vary widely implying that CPP derives from different structures in different individuals and sexes suggesting a multifactorial source of pain, including the pejorative explanation that it is “functional” [3–7]. Costs associated with CPP are high. In 1996 it was estimated that $881.5 million dollars were spent annually in the United States on outpatient visits alone [1]. CPP affects 15 to 24% of adult women [1,2]. These women routinely consult gynecologists for evaluation often without any gynecological abnormalities being found on examination. One type of CPP is pregnancy-related and 2–5% of all parous women experience disabling dysfunction two years after childbirth [8]. This is a global women’s health issue [9–13].

Referred pain is a well-known phenomenon that may occur in any pain condition [14–17]. It is defined by the International Association for the Study of Pain (IASP) as “pain perceived at a location that is not the origin of the pain” [18,19]. Referred pain can be provoked from tendons, ligaments, visceral and skeletal structures as well as from myofascial structures. Referred pain patterns elicited from skeletal and myofascial tissue has been studied from the beginning of 19^th^ century in different settings [14,16,20,21]. A sclerotome is defined as an area of bone and periosteum supplied by a single spinal segment [22], in contrast to dermatome and myotome as areas of skin and muscle supplied by a single spinal segment. Sclerotome charts have been reported in humans [15,23,24] but more extensively in rats [17]. In terms of location, there is considerable location discrepancy between dermatomes and sclerotomes particularly in the body trunk and proximal limbs [25]. Knowledge of these patterns can assist in understanding the source of CPP in women.

Pain mapping is a method to investigate the distribution of referred pain. The methodology has been used previously for identifying sclerotomal innervation and also for the evaluation of CPP [15,26,27]. Validation of pain mapping has been assessed with a systematic review [28]. The pelvic ligaments, muscles and/or their insertions have been proposed as sources of CPP [5,6,29]. The distribution of referred pain patterns provoked from intra-pelvic structures has, as far as we know, not previously been comprehensively described in women with CPP. This knowledge may be of value for the differential diagnosis of CPP.

The aim of this study was to describe the referred pain patterns provoked from 13 predetermined intra-pelvic anatomical landmarks in parous women with CPP and contrast those patterns with those provoked in parous women without CPP. The primary outcome was the referred pain distribution as indicated in pain maps and the secondary outcome was the size of the referred pain areas. The hypothesis tested was that a light manual pressure to intra-pelvic structures could provoke referred pain in women with CPP over and above that of women without CPP.

## Materials and Methods

### Study population

A cohort of 36 parous women with CPP was included; they have been described in detail elsewhere [5]. Briefly, the women were recruited from a physiotherapy department waiting list and by advertisements in newspapers. The inclusion criteria were: 1) reporting pain in the sacral region (buttocks included) with onset during a pregnancy and persisting at least six months after delivery, 2) reporting present pain intensity above 30 mm on a visual analogue scale (VAS) where 0 mm is no pain and 100 mm is worst possible pain, 3) having at least one of three positive pelvic pain provocation test, 4) having ipsilateral pain elicited on internal palpation at the ischial spine and 5) ability to understand Swedish. Exclusion criteria were: 1) reporting persistent pain in the back or pelvis with onset prior to pregnancy, 2) previous back surgery and 3) positive straight leg raising test or loss of a tendon reflex in a lower extremity.

In order to recruit a control group of parous women without CPP, 44 women from an organized gynecological screening at a midwifery surgery in a Primary Health Care Centre were consecutively assessed for eligibility. For blinding purposes *i*.*e*. blinded for those who performed the pain mapping procedure this group was a mix of women with and without low back and pelvic pain who were both parous and non-parous. An initial assessment procedure was carried out by a physiotherapist not involved in the pain mapping procedure. After the initial assessment and the pain mapping procedure 15 women were excluded (12 women with low back or pelvic pain and three non-parous) according to the inclusion criteria: 1) having given birth at least once but not within the last six months, 2) no reported low back or pelvic pain on a pain drawing or elicited by pain provocation tests and 3) ability to understand Swedish. Thus, the control group consisted of 29 women, henceforth denoted as women “without CPP”.

### Initial assessment procedure

**Fig 1:**
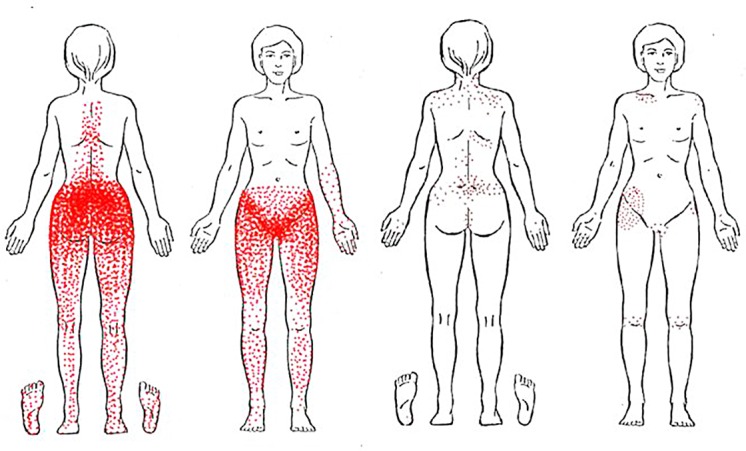
Distribution of reported pain at study inclusion among women with and without chronic pelvic pain. Women with CPP are presented on the left side and those without CPP on the right side.

The initial assessment procedure consisted of a questionnaire and an external physical examination. The questionnaire included a pain drawing of the body (Fig. 1), questions about the time of onset of any ongoing low back or pelvic pain, number of previous deliveries, date of latest delivery, cigarette smoking at present (yes/no) and educational level (≤12 years/>12 years). On the pain drawing the women indicated any location of pain. More than one location could be indicated. Furthermore, the women were requested to report the pain intensity at present and worst pain during the past week using VAS, which ranged from 0 (no pain) to 100 mm (worst possible pain) [30]. They also completed the Disability Rating Index questionnaire (DRI), an instrument for self-reported physical function [31] (0–100 mm) where lower values represent higher function.

The external physical examination of the pelvis and back included pain provocation tests, which were considered positive if they provoked pain in the pelvis or back, otherwise negative and were used to discriminate women with CPP from women without CPP. The pain provocation tests of the pelvis, i.e. Menell’s, Patrick’s and Posterior Pelvic Pain Provocation (P4), were used to aggravate ipsilateral sacral (buttocks included) pain and were performed on each leg with the women in the supine position [32,33]. The provocation tests of the back were maximum flexion/extension while standing and paravertebral palpation of the low back and iliolumbar ligaments. In testing for neurological signs, test of reflexes and straight leg raising (SLR) was used. Loss of patellar or Achilles tendon reflexes was considered positive. SLR was tested passively in each legs with the women in the supine position and was considered positive if neurological symptoms occurred or radiating pain was provoked [34].

### Pain mapping procedure

**Fig 2:**
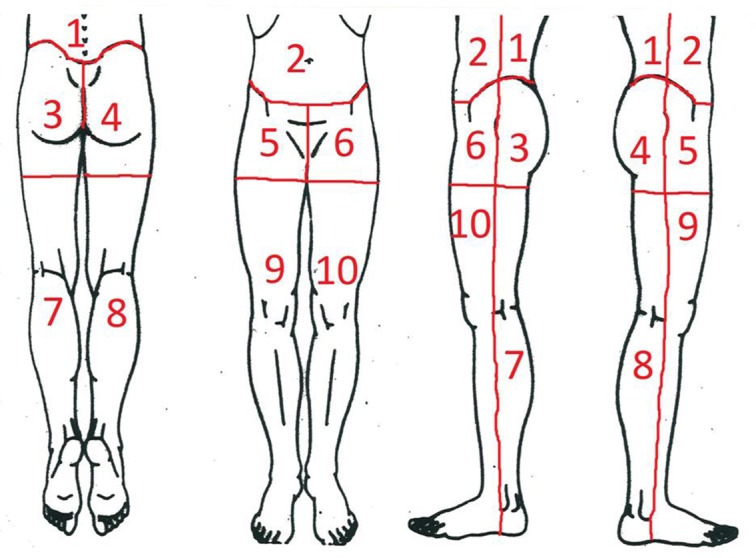
Cartoon model with marked body regions used in pain mapping procedure. 1) lumbar, 2) abdomen, 3) sacral left side, 4) sacral right side, 5) groin left side, 6) groin right side, 7) leg back left side, 8) leg back right side, 9) leg front left side, and 10) leg front right side.

**Fig 3:**
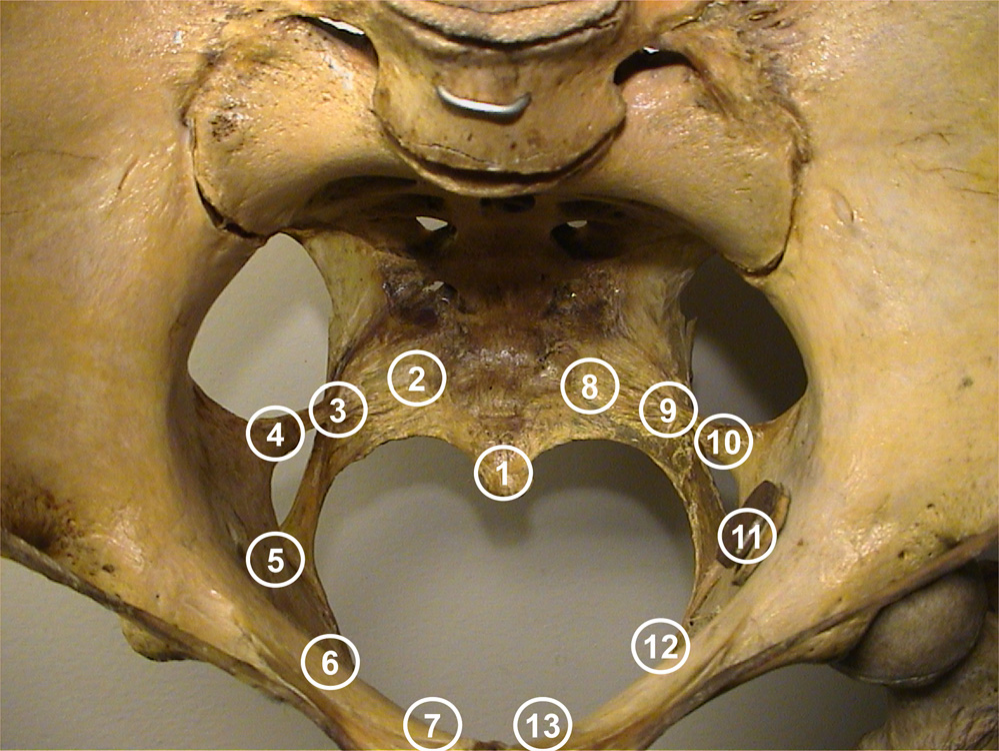
Thirteen predetermined intra-pelvic anatomical landmarks. 1) os coccyx, 2 and 8) os sacrum laterally, 3 and 9) sacrospinous ligament, 4 and 10) ischial spine, 5 and 11) os ischii, 6 and 12) os pubis laterally and 7 and 13) os pubis medially.

Pain mapping was here defined as the perceived pain distribution provoked by vaginal palpation of 13 predetermined intra-pelvic anatomical landmarks and expressed in separate pain drawings of the lower part of the body, as shown in Fig. 2 (body region borders and their numbers, used in the analyses, were not shown to the women). The predetermined anatomical landmarks were: the coccyx, the lateral part of sacrum at the insertion of the sacrospinous ligament, the middle part of the sacrospinous ligament, the insertion of the sacrospinous ligament at the ischial spine, the ischium inferior to the ilio-ischial fusion and the lateral and medial part of the pubic bone (Fig. 3). All the landmarks were examined bilaterally except the coccyx and all in the same order in all women. The vaginal palpation was performed by a physician (P.K.) and the women’s recording in pain drawing was aided by a physiotherapist (T.T.).

The vaginal palpation of the intra-pelvic landmarks was performed with the women in the supine lithotomy position. Pain provocation was by a light manual pressure on each of the landmarks and the women were asked to draw the distribution of perceived pain or other sensation on a cartoon and report the pain intensity on a Likert scale 0–2, (0 = no pain, 1 = moderate pain and 2 = intensive pain), resulting in a total of 845 pain drawings (65 women x 13 landmarks). They were also told to freely express their experience verbally and this was afterwards categorized by four descriptors: “Blank” = no sensation at all, “Other” = non-painful referred sensations, “Diffuse” = hard to draw in pain drawing and “Distinct” = well defined pain in pain drawing (Table 1).

**Table 1:** Number (%) of women with perceived sensation provoked on 13 intra-pelvic anatomical landmarks and referred to different body regions^1)^, expressed in pain drawings among women with and without chronic pelvic pain (CPP).

Body region	With CPP (n = 36)				Without CPP (n = 29)			
	Blank	Other	Diffuse	Distinct	Blank	Other	Diffuse	Distinct
None	0				2 (6.9)			
Lumbar		1 (2.8)	1 (2.8)	7 (19.4)		3 (10.3)	0	0
Abdomen		1 (2.8)	6 (16.7)	2 (6.9)		0	1 (3.4)	
Sacral left		7 (19.4)	11 (30.6)	35 (97.2)		25 (86.2)	0	15 (51.7)
Sacral right		5 (13.9)	14 (38.9)	36 (100)		26 (89.7)	0	12 (41.4)
Groin left		6 (16.7)	10 (27.8)	32 (88.9)		24 (82.8)	1 (3.4)	16 (55.2)
Groin right		4 (11.1)	12 (33.3)	33 (91.2)		20 (69.0)	3 (10.3)	11 (37.9)
Leg back, left		0	0	2 (5.6)		0	0	0
Leg back right		0	0	1 (2.8)		0	0	0
Leg front left		0	0	1 (2.8)		0	0	0
Leg front right		0	0	1 (2.8)		0	0	0

“Blank” = no sensation, at all, “Other” = non-painful referred sensations “Diffuse” = hard to draw in pain drawing, “Distinct” = well defined pain in pain drawing.

^1)^ Refers to Fig. 2.

### Composite pictures and area calculation

All pain drawings from the initial assessment (n = 65) were accounted for. Pain drawings from the pain mapping procedure were taken into consideration according to the Likert scale. After exclusion of pain drawings without indicated pain (Likert scale 0) 421 of 468 pain drawings from women with CPP and 111 of 377 from women without CPP remained. All remaining pain drawings were scanned to images of 250 millimetres from head to toe and 50 millimetres from hip to hip.

To produce composite pictures each scanned pain drawing was digitally transformed by replacing the markings on the cartoon with approximately one dot (12 pixels) per mm^2^ with use of Adobe Photoshop. Subsequently, the digitalized pain drawings from all women were superimposed to get composite pictures. This resulted in one composite picture from the initial assessment and 13 composite pictures i.e. pain maps from the vaginal palpation representing each of the landmarks.

The area of provoked pain distribution was calculated by using Image Measurement. On every scanned image the drawn pain area was manually outlined. Subsequently the program calculated the outlined area on the cartoon in mm^2^. Pain marked by the women as a cross in pain drawings was transformed to a circular area with the smallest arm of the cross as the radius.

### Statistical analysis

Summary statistics were computed using standard methods and presented as medians and numbers. Non-parametric tests were used to compare differences between and within the groups and consisted of Fischer’s exact test for characteristic variables, Wilcoxon’s test for continuous variables and signed rank test for dependent continuous variables. In correlation analyses Spearman’s rank order correlation coefficient was used. P-values less than 5% were regarded as statistically significant. Very small p-values were indicated as <0.0001. Study size calculation was not performed. Statistical analysis was performed using the SAS program package version 9.3 (SAS Institute, Cary, NC).

### Ethical statement

Ethical approval was granted by the medical ethics committee of Umeå University, Sweden and all participants gave written informed consent.

## Results

The number of previous pregnancies and deliveries were similar between the groups. Women with CPP reported higher pain intensity, were younger, and had lower physical functioning (Table 2).

**Table 2:** Characteristics of women with and without chronic pelvic pain (CPP). Figures are medians (25^th^ to 75^th^ percentiles) and numbers (%).

Characteristic	With CPP n = 36	Without CPP n = 29	P
Age (yr)	32.2 (29.0 to 37.1)	44.0 (38.3 to 46.6)	<0.0001
No. of previous pregnancies	2 (2 to 3)	2 (2 to 3)	0.69
No. of previous deliveries	2 (2 to 2)	2 (2 to 2)	0.99
Time since last delivery (yr)	2.1 (1.4 to 3.3)	8.0 (4.3 to 16.3) ^1^ ^)^	<0.0001
No. of deliveries before onset of pain	1 (1 to 2)	Not applicable	-
Duration of pain (yr)	4.2 (2.4 to 6.4)	Not applicable	-
Pain intensity, at present (mm) ^2^ ^)^	34.0 (27.5 to 50.5)	0 (0 to 0)	<0.0001
Pain intensity, as worst past week (mm) ^2^ ^)^	59.5 (43.0 to 75.0)	0 (0 to 8.0)	<0.0001
Disability rating index (mm) ^2^ ^)^	53.3 (38.0 to 66.5)	1.9 (0 to 6.4)	<0.0001
Hormonal contraceptive use (%)	9 (25.0)	10 (37.0)	0.41
No cigarette smoking (%)	32 (88.9)	24 (92.3)^3^ ^)^	1.0
Education >12 years (%)	16 (44.4)	16 (61.5)^3^ ^)^	0.21

^1)^ n = 22

^2)^VAS, Visual Analogue Scale

^3)^ n = 26

High density pain distribution was displayed in the sacral, buttock, groin and symphyseal areas and lower density pain distribution in the thoracic and lumbar back, lower abdominal areas and the legs among women with CPP. Low density pain distribution was shown on the shoulders, low back, right hip and knees among women without CPP (Fig. 1).

**Fig 4:**
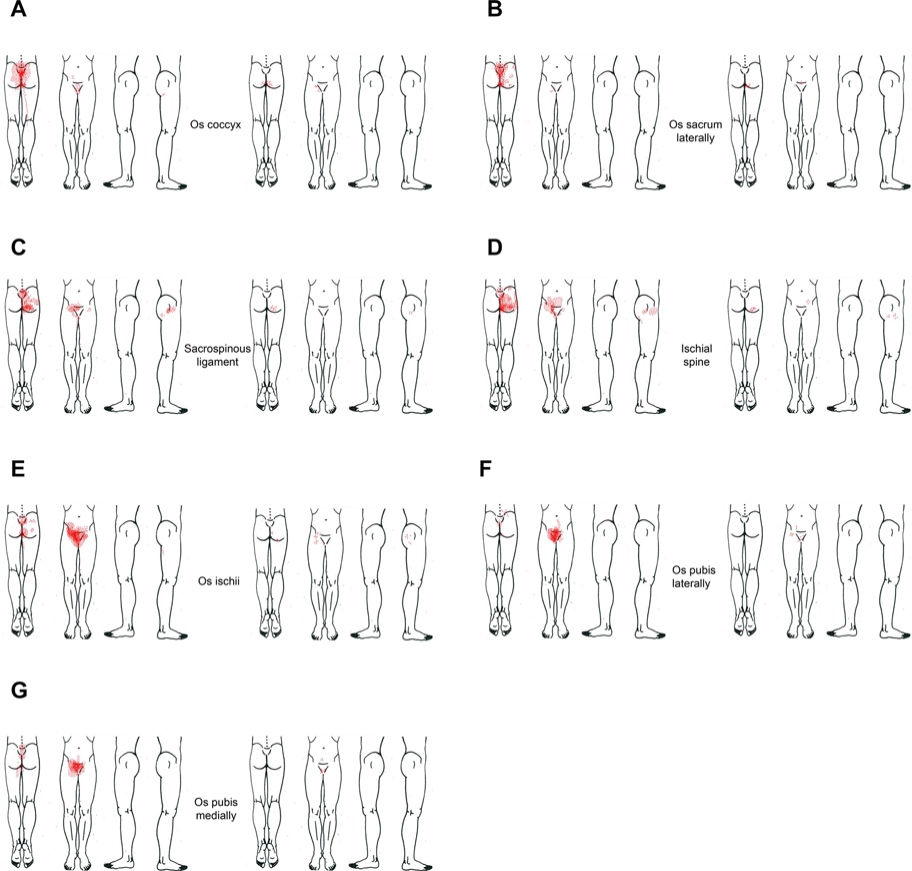
Distribution of referred pain provoked by palpation at the respective intra-pelvic anatomical landmark. Palpation on the right side and coccyx, among women with and without chronic pelvic pain. Women with CPP are presented on the left side and those without CPP on the right side, in every figure.

Distribution of pain provoked from the right-sided intra-pelvic landmarks including the coccyx among women with and without CPP is displayed on the pain maps (Fig. 4a-g). For both groups the referred pain patterns from the right side were almost identical to those from the left side (data not shown). In general, pain provoked by palpation on the posterior intra-pelvic landmarks was mostly referred to the sacral and buttock regions, and pain provoked by palpation on the lateral and anterior intra-pelvic landmarks was mostly referred to the groin and pubic regions, with or without pain being referred down the ipsilateral leg.

Intra-pelvic provoked sensations were mainly confined to the sacral and the groin regions, where diffuse and distinct referred pain was mostly experienced by women with CPP while non-painful referred sensations were mostly experienced by women without CPP (Table 1). Provoked sensations from the lower back and abdomen showed similar differences although with lower frequency. Sensations to the legs was perceived only by a few women with CPP and limited to distinct pain.

Of the 845 pain drawings from the vaginal palpation “no sensation at all” or “non-painful referred sensations” were provoked in 9% and 71%, respectively, among women with and without CPP, (data not shown).

The average provoked pain distribution area (mm^2^) from all 13 anatomical landmarks was approximately tenfold in women with CPP as compared to women without CPP, p<0.0001 (Table 3). The largest pain distribution areas were provoked on the ischial spine and on the ischium inferior to the ilio-ischial fusion, with decreasing areas in both directions further away from these landmarks, <0.0001 < p < 0.009, (data not shown).

**Table 3:** Pain distribution areas on pain drawings (mm^2^) provoked by palpation of 13 anatomical landmarks in the small pelvis, among women with and without chronic pelvic pain (CPP). The sum and average of all 13 anatomical landmarks, the average of symmetric landmarks and os coccyx are presented as medians (25^th^ to 75^th^ percentiles).

Anatomical landmark	With CPP (n = 36)	Without CPP (n = 29)	p
Sum of all 13 landmarks	394.2 (249.6 to 698.6)	41.3 (12.8 to 66.4)	<0.0001
Average of all 13 landmarks	30.3 (19.2 to 53.7)	3.2 (1.0 to 5.1)	<0.0001
Os sacrum laterally	18.4 (7.1 to 43.1)	0 (0 to 0)	<0.0001
Sacrospinous ligament	28.5 (17.6 to 44.8)	0 (0 to 3.8)	<0.0001
Ischial spine	42.1 (23.6 to 61.7)	0 (0 to 5.6)	<0.0001
Os ischii	38.2 (20.8 to 50.6)	4.4 (0 to 9.6)	<0.0001
Os pubis laterally	25.7 (12.4 to 49.9)	0 (0 to 4.2)	<0.0001
Os pubis medially	16.6 (8.6 to 45.8)	0 (0 to 2.4)	<0.0001
Os coccyx	22.8 (12.0 to 43.9	0 (0 to 0)	<0.0001

## Discussion

Referred pain patterns provoked from intra-pelvic landmarks in women with CPP are consistent with sclerotomal sensory innervation and the magnification of the patterns in those with CPP over those without indicates central sensitization. The hypothesis that a light pressure to intra-pelvic structures could provoke referred pain patterns in women with CPP was proven. Pain location elicited by history and the location of provoked pain was similar among women with CPP whereas women without CPP experienced only minor, localized discomfort when intra-pelvic landmarks were provoked. Although this was a limited evaluation of sclerotomal structures within the pelvis, it provides strong evidence that pain can be generated from non-visceral intra-pelvic structures and that these are probable origins of at least a part of the symptoms of women with CPP. In the case of those sufferers of CPP where no visceral pathology can be found, this finding offers a new explanation.

The pain mapping procedure in this study showed a clear difference in the size of the areas of referred pain between the subjects with and without CPP. An interesting finding was that there was a close similarity in the location and size of the areas provoked by pressure in the CPP cohort with the sclerotomal mapping done by Inman and colleagues in the ‘40’s [15]. Inman et al used various nociceptive stimuli with the intent to elicit referred pain and use the referred pain areas to identify the nervous innervations of sclerotomal structures. That study concluded that the referred pain pattern indicated that the sclerotomal structures were innervated by spinal nerves subserving the cutaneous area mapped during stimulation. In comparing the similarities in referred pain patterns between Inman et al and this study, it became obvious that a mild pressure stimulus in the CPP subjects had similar effects to noxious stimuli in the volunteers used by Inman, himself included, who had no chronic pain. This is indirect evidence for central sensitization in the women with CPP who demonstrate allodynia to light pressure on intra-pelvic sclerotomal structures and expanded receptor fields in the referral areas. Referred pain patterns from pelvic floor muscles have been studied by Travell and Simons [20], and are somewhat similar to the distribution of referral patterns demonstrated in this study, but there are also major differences implying another pain mechanism in our CPP subjects than that originating only from muscles. When provoking the internal female genital organs, pain responses are vague, both in intensity and location, and contrary to our findings, not referred to the low back or thigh [35]. As with visceral stimulation, the participating women, with or without CPP, also could not recognize the precise site of the provocation.

A majority of studies show that CPP increases with age [36]. In an English study the age group with the lowest incidence of CPP was in age groups 18 to 25 years and 31 to 35 years, whereas the highest was in the 36 to 44 years olds [37]. Given this data, the clear difference between our CPP and control groups is even more striking than the statistical data shows.

### Possible explanations and implications

Referred pain patterns are common phenomena with complex backgrounds that have both central and peripheral mechanisms depending on the source. There are some theories with some experimental support [16], and there is consensus in the fact that referred pain exists and can be useful in diagnostics. One well accepted theory to explain the existence of referred pain is the physiological fact of convergence and divergence of peripheral nerve fibres to and from the spinal cord [38,39]. Convergence refers to the fact that a single dorsal horn cell can receive input from a wide variety of structures over a large area of the body [40]. Divergence refers to the fact that information from a restricted area in the periphery can enter the spinal cord at several different levels.

Peripheral and central sensitization of the nervous system can contribute to an expansion of the receptor fields of dorsal horn neurons and also to allodynia in the periphery. Central sensitization is a major contributor to persistent and chronic pain of various etiologies and is described as “pain hypersensitivity by changing the sensory response elicited by normal inputs” [41]. Receptor field expansion by priming the sensory system with acute pain has been demonstrated by studies done by Gillette, Kramis and Roberts [40]. Although theirs was an acute animal model, it showed that the onset of acute pain can expand the receptor fields of single dorsal horn neurons when musculoskeletal structures are stimulated by mildly noxious stimuli.

The women with CPP responded to mild, focal pressure on sclerotomal structures in the pelvis with both a perceived increase in the area of referred pain and an increased level of perceived pain as compared with controls without CPP. The hypothesis here is that an acute pain stimulus under pregnancy and/or during delivery had the same effect to “prime” the somatosensory system as in the Gillette et al. study but that effect persisted after the resolution of the acute event. Why some individuals continue to experience pain and others do not is the subject for further studies: there is not enough evidence from the current evaluation of the two populations to allow a hypothesis to be generated. Since the usual pain in the women with CPP is provoked by ordinary non-noxious activities that stimulate intra- and extra-pelvic structures such as prolonged sitting, prolonged standing and walking, sensitization must be inferred.

One can debate which type of tissue is the pain generator in CPP but the importance of this study is that it provides proof that non-visceral structures can contribute to CPP for a group of women with significant suffering and limitations in daily living. If the pain is provoked from muscles, connective tissue or skeletal structures or a combination, this is not an issue for the patient unless the treatment differs depending on the source.

### Strengths and weaknesses

To our knowledge this is the first study to produce pain maps and explore the importance of intra-pelvic structures in relation to CPP. The diagnostic method was safe and did not give either of the groups any adverse effects except for the unpleasantness from the vaginal examination approach. The use of pain drawing cartoons to construct composite pictures of referred pain patterns was a methodological strength as was the inclusion of women without CPP where the examiner was blinded as to those with and without CPP. The presence of possible co-morbidities that might be involved in the pain mechanism is a limitation in the study even if serious illness was excluded and visceral pathology had been ruled out. Also, intra-pelvic structures other than those provoked in the present study could be involved as pain generators but this was not tested. If so, the mechanism can be assumed to be similar but treatment might be different. The light pressure on each anatomical landmark was meant to be equal and the use of an instrument to measure the pressure [42] was taken under consideration but not chosen because of the difference in resistance of the various tissues inside the pelvis, i.e. periosteum v.s. ligament. Intra-examiner variability cannot be overlooked but the blinding procedure is against this.

Pain mapping by stimulation of non-visceral intra-pelvic structures can contribute to the diagnosis of CPP and thereby reduce costs and risks from other, sometimes costly and possibly harmful, investigations and treatment.

Future studies should focus on testing the accuracy of pain mapping in other groups with low back and pelvic pain, for example patients with verified disc herniation, patients with chronic prostatitis and patients with painful endometriosis. Similar allodynia and augmented receptor fields are likely. If it is possible to establish specific pain patterns from different conditions it would be beneficial for both patients and therapists.
